# The effects of dopamine receptor 1 and 2 agonists and antagonists on sexual and aggressive behaviors in male green anoles

**DOI:** 10.1371/journal.pone.0172041

**Published:** 2017-02-10

**Authors:** Alexandra N. Smith, David Kabelik

**Affiliations:** 1 Department of Biology, Rhodes College, Memphis, Tennessee, United States of America; 2 Program in Neuroscience, Rhodes College, Memphis, Tennessee, United States of America; University of Cologne, GERMANY

## Abstract

The propensity to exhibit social behaviors during interactions with same-sex and opposite-sex conspecifics is modulated by various neurotransmitters, including dopamine. Dopamine is a conserved neurotransmitter among vertebrates and dopaminergic receptors are also highly conserved among taxa. Activation of D_1_ and D_2_ dopamine receptor subtypes has been shown to modulate social behaviors, especially in mammalian and avian studies. However, the specific behavioral functions of these receptors vary across taxa. In reptiles there have been few studies examining the relationship between dopaminergic receptors and social behaviors. We therefore examined the effects of D_1_ and D_2_ agonists and antagonists on sexual and aggressive behaviors in the male green anole lizard (*Anolis carolinensis)*. Treatment with high doses of both D_1_ and D_2_ agonists was found to impair both sexual and aggressive behaviors. However, the D1 agonist treatment was also found to impair motor function, suggesting that those effects were likely nonspecific. Lower doses of both agonists and antagonists failed to affect social behaviors. These findings provide some evidence for D2 receptor regulation of social behaviors, but in contrast with previous research, these effects are all inhibitory and no effects were found for manipulations of D1 receptors. A potential reason for the lack of more widespread effects on social behaviors using moderate or low drug doses is that systemic injection of drugs resulted in effects throughout the whole brain, thus affecting counteracting circuits which negated one another, making measurable changes in behavioral output difficult to detect. Future studies should administer drugs directly into brain regions known to regulate sexual and aggressive behaviors.

## Introduction

Animal social behaviors are regulated by a social behavior neural network that interacts with the mesolimbic reward system to form a social decision-making network [1,2]. The catecholamine dopamine is one of the primary signaling molecules that regulates activity within this neural network. Dopamine is known to be involved in motivation and reward-seeking behaviors [3], locomotion [4], and social behaviors, including sexual [5] and aggressive [6] behaviors. Established dopamine-mediated social behaviors include sexual behavior in birds [7–9], mammals [10–12], and reptiles [13,14], as well as aggressive behaviors in mammals [15,16] and birds [17,18]. However, dopamine’s involvement in sexual and aggressive behaviors across species is varied in its effects [14,17–20].

Reptiles are a taxonomic group that has been largely overlooked in studies on dopamine’s involvement in sexual and aggressive behaviors; however, they are a potentially important model for examining the effects of dopamine on social behaviors, since they are the closest living relatives to birds and mammals [21,22]. Previous research suggests that the structure of the dopaminergic system exhibits a conserved pattern among reptiles [21], as well as across amniotic vertebrates [23]. However, it is unknown whether the functions of this dopaminergic system are similar across taxa.

Only minimal evidence currently exists in lizards for causal dopaminergic regulation of social behaviors. In terms of aggression, green anole (*A*. *carolinensis*) males fed crickets injected with the dopamine precursor L-3,4-dihyrdoxyphenylalanine (L-DOPA) were found to possess higher central dopamine (but not serotonin or norepinephrine) concentrations and to concurrently exhibit lower aggression scores than control subjects [24]. Also in green anoles, extracellular dopamine levels increased in the nucleus accumbens and amygdala in males after exposure to a mirror, which the animals perceive as a social challenge from another invading male [25]. Similarly, green anole males that were paired with a perceived dominant individual showed increased dopamine in the raphe and amygdalar regions, whereas males paired with perceived subordinate individuals exhibited increased dopamine in the striatum, nucleus accumbens, hypothalamus, and midbrain tegmentum [26]. Further evidence linking dopamine with social behaviors was provided by a study of male brown anoles (*Anolis sagrei*), which detailed various populations of neurons immunoreactive for tyrosine hydroxylase, an enzyme involved in dopamine synthesis [27]. The percentage of these neurons colocalizing Fos, a protein used as an indicator of neural activation [28], correlated with the frequency of courtship and aggressive behaviors in a number of dopaminergic cell populations [27]. Catecholamines in the peripheral nervous system are also involved in social behaviors in lizards: the postorbital patch of skin behind the eye of a male green anole, known as an eye spot, darkens as a display of and response to aggression due to an increase in plasma catecholamine levels [29].

Most studies addressing dopamine’s causal effects on social behavior focus on activation or inactivation of the D_1_ and D_2_ receptors subtypes. The little direct evidence that exists for dopaminergic regulation of lizard social behavior is limited to the effects of D_1_ agonists and antagonists on sexual behavior in three lizard species: two related species of whiptail lizards (*Cnemidophorus inoratus* and *C*. *uniparens*) and leopard geckos (*Eublepharis macularius)* [13,14]. In whiptail lizards, systemic injection of a D_1_ agonist increased mounting behavior [14], while in leopard geckos, systemic injection of a D_1_ antagonist inhibited courtship behavior [13]. However, in the whiptail lizard study, the D_1_ agonist doses that caused a significant change in behavior were extremely small at 0.005 (in the hybrid triploid species) and 0.05 μg/mg (in the parental diploid species) [14]. Additionally, only consummatory sexual behaviors (i.e. mounting and copulation), and not appetitive sexual behaviors (i.e. anticipatory behaviors) were examined in this study. Appetitive courtship behaviors were examined in the leopard geckos experiment, but these data are unpublished and only referred to in a review, and they involve fairly high doses of D_1_ antagonists (4–8 mg/kg) [13]. The effects of D_2_ receptor activation on lizard social behaviors are completely unknown. We hypothesize that D_1_ and D_2_ receptor activation modulates both sexual and aggressive behaviors in male green anoles because these receptors play such roles in other amniote species. We predict that the activation of D_1_ receptors will have similar effects as seen in other species [18,19,30,31], specifically, that a D_1_ agonist will increase sexual and aggressive behaviors in male green anoles. However, while D_2_ receptor activation has been shown to be involved in sexual and aggressive behaviors in other species, the effects do not alter behavior in a consistent manner [17–20,32,33]. Therefore, we predict that the D_2_ agonist will have an effect, but we do not make a prediction on the directionality of this effect.

## Methods

### Subjects

The subjects used in this experiment were male green anoles (*Anolis carolinensis*). They were purchased from Sullivan Amphibians in Nashville, TN, and housed on a 14:10 hour light-dark schedule, and a temperature range of 24–31°C (temperature peaking at mid-day), with additional heat provided by a 60-watt light bulb suspended above half of each terrarium (30.5 cm H x 26 cm W x 51 cm L). The preferred daytime body temperature of these lizards is between 30–34°C [34]. The focal males were housed individually, while the stimulus males were each housed with two stimulus females. All the focal males were kept in visual isolation from each other with an opaque divider between terraria. All animals were monitored daily and fed live crickets three times a week. Five animals died of natural causes during the course of these experiments and were excluded from analyses. All procedures were approved by the Rhodes College Institutional Animal Care and Use Committee (protocol 101) and are in accordance with federal guidelines.

### Behavioral testing

Testing was conducted during breeding-condition periods between June 2014 and April 2016. Each focal male was sized-matched with a stimulus male based on snout-vent length, with the stimulus male being no more than 0.2 cm longer or shorter than the focal male. The aggressive and sexual display behaviors (Table 1) examined in this study were the same as those observed in other studies investigating courtship and aggression in lizards [14,27,35].

**Table 1:** An ethogram of behaviors recorded for the focal males and stimulus animals during behavioral trials.

Behaviors	Description
Head Bob^A^^,^^B^	Nodding up and down of the head, while the rest of the body remains immobile, with each occurrence differentiated by a slight pause
Push Up^A^^,^^B^	Lifting up and down of the entire body, with each occurrence differentiated by a slight pause
Dewlap Extension^A^^,^^B^	A full extension of the dewlap (throat fan)
Dewlap/Push Up^A^^,^^B^	Combined execution of Dewlap Extension and Push Up, with each occurrence differentiated by a slight pause
Chase^A^^,^^B^	Rapid pursuit of a conspecific
Bite^B^	Physical contact in the form of a bite (only occurred in male-male trials)
Copulate^A^	Copulation with the conspecific (only occurred in male-female trials)
Dorsal Crest	Extension of the dorsal crest
Eye Spot	Darkening of the postorbital skin

^A^ Behaviors were summed to obtain total frequency of sexual behaviors.

^B^ Behaviors were summed to obtain total frequency of aggressive behaviors.

All drugs were dissolved in 0.9% NaCl, and administered intraperitoneally at a volume of 0.05 mL, 30–60 min prior to behavioral testing. Repeated-subjects experiments were run with subjects given at least two-week breaks between different treatments. The two-week gap between testing minimized the possibility of recognition between the male anoles as male green anoles exhibit recognition of opponents of aggressive encounters for seven to ten days [36]. Within experiments, the same focal males were also always paired with the same stimulus males to minimize behavioral variation between drug and saline treatments.

For the courtship trials, 2 females were placed in the focal male’s terrarium and the behaviors of both stimulus females and the focal male were recorded for 10 minutes. Once the females were removed, a mirror aggression trial was run by placing a mirror on the outside of the focal male’s terrarium opposite of the heat lamp and opposite the top of the perch, and behaviors were again recorded for 10 minutes. The mirror trial was included to test for the initiation of aggressive behavior; the animals do not recognize their reflection, so the focal males view the image as an intruding male [25]. The mirror was then removed and a stimulus male was placed in the focal male’s terrarium for the aggression trial, and both focal and stimulus males’ behaviors were recorded for 10 minutes. These same behavioral trial methods were used in all experiments.

### Drugs

The D_1_ agonist SKF 38393 (Sigma-Aldrich, St. Louis, MO, USA; catalog item DO47) was injected at 10, 1, 0.1, 0.01, 0.001 mg/kg and 0.05 and 0.005 μg/kg doses. The 1 mg/kg dose is a standard dose that has achieved effects on social behaviors in other species [17,19,30]. The 0.1, 0.01, and 0.001 mg/kg doses were chosen to test the effects of small doses akin to those used by Balthazart et al. [19]. The 0.05 and 0.005 μg/kg doses were chosen to test the effects of even smaller doses, akin to those used by Woolley et al. [14]. The D_2_ agonist quinpirole (Sigma-Q111) was injected at 10 and 1 mg/kg doses. The D_1_ antagonist SCH-23390 (Sigma-Aldrich, St. Louis, MO, USA; catalog item D054) was injected at 1 and 0.1 mg/kg doses. The D_2_ antagonist raclopride (Sigma-R121) was injected at 1 and 0.1 mg/kg doses.

A small follow-up D_1_ agonist study was also run to test the repeatability of a significant result obtained for the 0.001 mg/kg D_1_ agonist dose in the mirror aggression trials.

### Data analysis

All analyses utilized non-parametric tests because the data did not meet the assumptions of parametric tests. The frequencies and latencies of social behaviors following drug or saline treatment were analyzed using Wilcoxon Signed-Rank tests or Friedman non-parametric repeated measures ANOVA, with significance set at p < 0.05. Post-hoc analyses were conducted using Wilcoxon Signed-Rank tests. Instead of adjustments for running multiple tests, we ran follow-up repeatability experiments on significant results.

## Results

### D_1_ agonist

**Fig 1:**
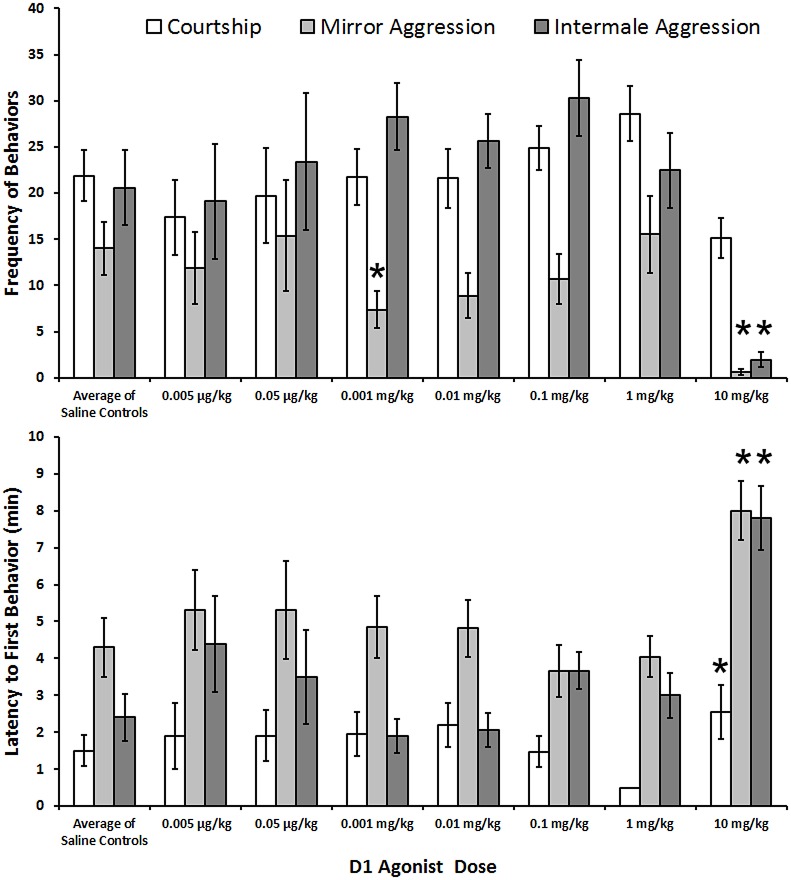
A dose response graph of the frequencies of (Top) and latencies to first display (Bottom) of sexual and aggressive behaviors in experiments using the D_1_ agonist SKF 38393. The control values were averaged from all experiments using D_1_ agonists. The error bars represent ± S.E.M. The * represents significance of p < 0.05 compared to control.

There were significant differences found in the 10 mg/kg treatment with SKF 38393 relative to control (N = 20). Behavioral frequencies (Z = -3.62, p<0.001, Z = -3.92, p<0.001) and latencies (Z = -3.01, p = 0.003, Z = -3.57, p<0.001) were both affected in the mirror trial and intermale aggression trials, respectively. Fig 1 depicts results from experiments using the D_1_ agonist. Additionally, the latency (Z = -2.41, p = 0.016), but not frequency (Z = -1.06, p = 0.29), of behaviors in the courtship trial was also significantly greater following drug treatment relative to saline. A transient cessation of all locomotor activity at approximately 50 minutes was noted following this high agonist dose and subjects remained in a state of very low motor activity for a period of minutes to hours, but subsequently regained normal motor functions. Separate analyses of the frequencies and latencies of the dorsal crest and eye spot did not offer any additional insight into the role of D_1_ and D_2_ receptors on social behaviors, so those data are not reported for any experiment.

There were no significant differences (p>0.05 for all) in recorded behaviors following the D_1_ agonist treatment at the 1 mg/kg dose (N = 20).

Following 0.1, 0.01, and 0.001 mg/kg doses (N = 22), only one value was significant: the frequency of aggressive behaviors in the mirror trial was significantly lower in the 0.001 mg/kg dose than saline treatment (χ^2^ = 11.89, df = 3, p = 0.008; p>0.05 for all other comparisons). However, a follow-up replication of this behavioral analysis (N = 22) failed to find a significant difference between the 0.001 mg/kg (mean = 6.2, S.E. = 2.12) and saline (mean = 4.1, S.E. = 1.93) groups (Z = -1.34, p = 0.18).

Finally, there were no significant differences (p>0.05 for all) in recorded behaviors following treatment with the 0.05 and 0.005 μg/kg doses, relative to saline (N = 10).

### D_2_ agonist

**Fig 2:**
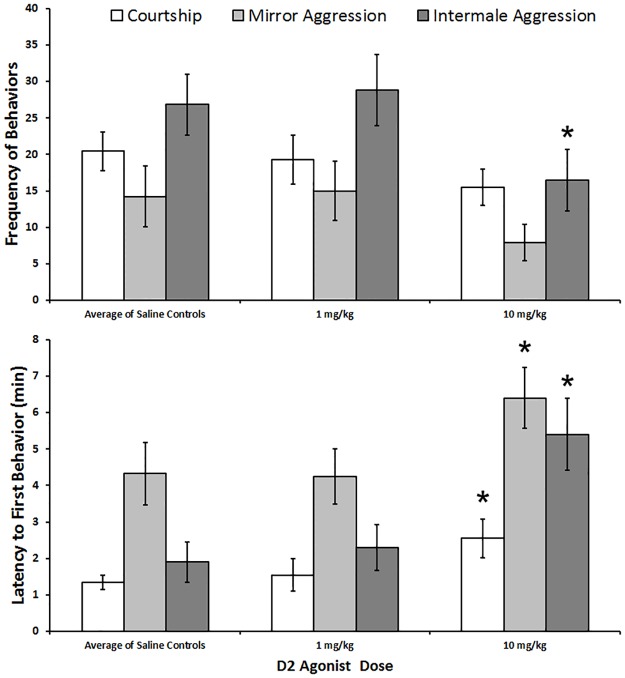
A dose response graph of the frequencies of (Top) and latencies to first display (Bottom) of sexual and aggressive behaviors in experiments using the D2 agonist quinpirole. The control values were averaged from all experiments using D2 agonists. The error bars represent ± S.E.M. The * represents significance of p < 0.05 compared to control.

The lizards treated with 10 mg/kg of the D_2_ agonist quinpirole (N = 20) displayed significantly fewer aggressive behaviors in the intermale trials than lizards treated with saline (Z = -2.29, p = 0.022; Fig 2). The latency to display courtship behaviors (Z = -2.57, p = 0.010), as well as aggressive behaviors in both the mirror (Z = -2.35, p = 0.019) and intermale aggression (Z = -2.74, p = 0.006) trials were greater when given the 10 mg/kg dose. There was no effect on behavioral frequency within courtship and mirror aggression trials. While there was no noticeable motor impairment as with the D_1_ agonist at 10.0 mg/kg, there was an increase in latency for all behaviors.

There were no significant differences (p>0.05 for all) in recorded behaviors following the D_2_ agonist treatment at the 1 mg/kg dose (N = 20).

### D_1_ antagonist

Following treatment with 1.0 and 0.1 mg/kg doses of the D_1_ antagonist SCH-23390 (N = 10), there were no significant differences or trends (p>0.05 for all) found between the behavioral frequencies and latencies of the males treated with saline and either drug dose (data not shown).

### D_2_ antagonist

As with the D1 antagonist, treatment with 1.0 and 0.1 mg/kg doses of the D_2_ antagonist raclopride (N = 10), there were no significant differences or trends (p>0.05 for all) found between the behavioral frequencies and latencies of the males treated with saline and either drug dose (data not shown).

## Discussion

Despite evidence for robust effects of D_1_ and D_2_ receptor activation on sexual and aggressive behaviors in other species, we were solely able to detect a general inhibition of social behaviors with high doses (10 mg/kg) of the D_2_ receptor agonist in male green anoles. An inhibition of social behaviors also occurred following high dose treatment with the D_1_ receptor agonist, but these were thought to be nonspecific effects given that they occurred concurrently with visible impairment of locomotion, thus making it impossible to separate social behavior effects from motor system effects. This cessation of locomotion lasted for a period of minutes to hours. Effects on motor function are unsurprising, given that both D_1_ and D_2_ receptors are involved in regulating motor function [4]. The D_2_ agonist did not qualitatively inhibit locomotion as did the D_1_ agonist. Therefore, the D_2_ effects on social behavior may be specific to social behavior circuitry. However, we must still be caution in accepting such an interpretation, as it is also possible that these high drug doses induced some nausea/discomfort that nonspecifically suppressed behaviors.

All lower doses of D_1_ and D_2_ agonists failed to affect any measure of social behavior, except for a decrease in the frequency of mirror trial aggression following the 0.001 mg/kg dose of the D_1_ agonist relative to saline treatment. Hence, we replicated this mirror trial as our multiple statistical comparisons may have resulted in false positive results due to chance. The replicated test failed to reproduce the original significant difference, suggesting that that difference had indeed arisen due to chance.

The lack of significant effects of the very low D_1_ agonist doses (0.005 and 0.05 μg/kg), which had previously been found to be effective in whiptail lizards, could be due to species differences or to the different type of agonists used between the studies [14]. The drug SKF 81297, used in Woolley et al. [14], is a full D_1_ receptor agonist with an efficacy rate comparable to dopamine itself [37]; however, SKF 81297 has now been found to act not only on D_1_ receptors but also partly on D_2_ receptors, as well as on D_1_-D_2_ receptor heterodimers, thus leading to changes in neural activity within target brain regions that differ from changes elicited by solely D_1_ receptor-specific drugs [38]. Perhaps the different results obtained here and in Woolley et al. [14] are therefore due to their use of a more promiscuous dopamine agonist. The presence of a relevant D_1_-D_2_ receptor heterodimer mechanism could also explain the lack of specific effects on social behavior in our study relative to manipulations of the whole dopaminergic system via L-DOPA treatment by Höglund et al. [24].

To further address our hypotheses, the effects of D_1_ and D_2_ antagonists were also examined in order to determine if a ceiling effect resulting from large amounts of endogenous dopamine release may have obscured potential roles of these receptors that were not apparent following agonist treatment. However, no significant effects on social behavior were found for either the D_1_ or D_2_ antagonist.

The lack of extensive effects of dopamine receptor manipulation on social behaviors in this study could be due to the peripheral administration of the drugs. Such an approach targets the entire brain and thus acts simultaneously on multiple brain regions that may all regulate social behaviors, but not all in the same manner. Simplistically, some regions may promote while others inhibit given behaviors, and thus one drug acting on both types of regions could cause effects that cancel each other out or at least create enough noise to impair detection of any actual effects. A more direct approach of drug delivery to brain regions involved in social behaviors might yield more telling results. For example, the nucleus accumbens, amygdala, prefrontal cortex, and parts of the hypothalamus are regions known to be involved in regulating aggression in mammals, but not all in the same manner [24,39–41]. And the release of dopamine in some of these regions during aggression encounters in green anoles appears to depend on whether the subject perceives that it is winning or losing an encounter [26]. A direct method of individually delivering dopaminergic drugs into these or homologous regions of the lizard brain might prevent the drug from binding to various counteracting brains regions and thus allow for the unmasking of further effects on social behavior. The case for direct administration of drugs to brain regions in future is bolstered by findings in the literature that detail differing effects of peripheral, intra-cerebroventricular, and brain-region-specific dopaminergic drug administration [9,42–44].
